# Gut microbiota: a new frontier in understanding and protecting endangered plateau schizothorax fish

**DOI:** 10.3389/fmicb.2025.1592312

**Published:** 2025-06-13

**Authors:** Hongbo Pan, Haiping Liu, Fei Liu, Jianmei Xie, Yan Zhou, Qize Zheng, Mingxiong Guo

**Affiliations:** ^1^College of Ecology and Environment, Tibet University, Lhasa, Xizang, China; ^2^Integrative Science Center of Germplasm Creation in Western China (CHONGQING) Science City, Key Laboratory of Freshwater Fish Reproduction and Development (Ministry of Education), Key Laboratory of Chongqing Municipality for Aquatic Economic Animal Resources Conservation and Germplasm Creation, School of Life Sciences, Southwest University, Chongqing, China; ^3^Integrative Science Center of Germplasm Creation in Western China (CHONGQING) Science City, Key Laboratory of Freshwater Fish Reproduction and Development (Ministry of Education), Key Laboratory of Chongqing Municipality for Aquatic Economic Animal Resources Conservation and Germplasm Creation, College of Fisheries, Southwest University, Chongqing, China; ^4^Aquaculture Science Research Institute, Tibet Academy of Agricultural and Animal Husbandry Sciences, Lhasa, Xizang, China; ^5^Key Laboratory of Southwest China Wildlife Resources Conservation College of Life Sciences, Xihua Normal University, Nanchong, Sichuan, China; ^6^College of Life Sciences, Wuhan University, Wuhan, Hubei, China

**Keywords:** middle reaches of the Yarlung Zangbo River, Xizang endemic fish, endangered fish, gut microbiota, 16S rRNA

## Abstract

**Introduction:**

Against the backdrop of global biodiversity decline, the role of gut microbiota in endangered species conservation remains underexplored. Endemic fish species in Xizang are critical to plateau ecosystems, yet many face severe survival threats. This study investigates the association between gut microbiota composition and conservation status in five endemic fish species, including the nationally protected *Oxygymnocypris stewarti, Schizothorax waltoni*, and *Schizothorax macropogon*.

**Methods:**

Using 16S rRNA sequencing, we systematically analyzed gut microbiota community structures across the five fish species. We compared microbial diversity, dominant bacterial phyla, and the influence of dietary habits on microbiota composition.

**Results:**

Dominant Bacterial Phyla: Fusobacteria, Proteobacteria, and Verrucomicrobia were common across species, while Tenericutes was uniquely dominant in endangered fish. Diversity Trends: Gut microbiota diversity followed the order: *Ptychobarbus dipogon* > *S. waltoni* > *Schizothorax o-connori* > *S. macropogon* > *O. stewarti*. Conservation Status Correlation: Species with higher endangerment levels exhibited significantly lower diversity: Least Concern (LC) > Near Threatened (NT) > Vulnerable (VU) > Endangered (EN). Dietary Influence: Phytophagous (PHY) fish had higher microbial diversity than omnivorous (OMN) and sarcophagous (SAR) fish, confirming diet as a key factor shaping gut microbiota.

**Discussion:**

This study provides the first evidence linking gut microbiota composition to the conservation status of endemic Tibetan fish. The reduced microbial diversity in endangered species suggests potential microbiome-related health vulnerabilities. Additionally, dietary differences significantly influence microbiota structure, highlighting the need for habitat and dietary conservation strategies. These findings open new avenues for microbiome-based conservation approaches in endangered species management.

## 1 Introduction

In vertebrates, a large number of rich microorganisms in the intestine aggregate to form a complex ecosystem that participates in the growth and development process of the host (Nayak, 2010; Walter et al., 2011). Similar to most vertebrates, fish also harbor a rich variety of gut microflora, including bacteria, viruses, fungi, protozoa, and microalgae (Yu, 2016). Fish, over time, adapt their gut microbiota to their ecological environment and dietary sources (Wong and Rawls, 2012; Miyake et al., 2015). However, variations in individual fish, diet, and life history inevitably lead to differences in gut bacterial colonization (Zhang, 2018). Studies have shown that food, by directly interacting with gut microbiota, forms a part of the microenvironment, thus directly influencing it (Huang et al., 2021). Some research further indicated that habitat, geographic distance, host evolutionary history, and diet are key factors affecting fish gut microbiota (Kim et al., 2021; Pan et al., 2023). Conversely, several studies have also found that the gut microbiota has multiple influences on the growth and diet of wild fish. For instance, Li Xuemei et al. discovered through metagenomic fingerprinting analysis and high-throughput sequencing of bacterial 16S rRNA genes that there were significant differences in the intestinal microbiota between transgenic carp and wild-type carp (Li et al., 2018), indicating that the intestinal microbiota is closely related to the growth of carp.

China is recognized as one of the major distribution areas for schizothoracin fishes in the world, known for its rich variety of species. Particularly in the Qinghai-Tibet Plateau, schizothoracin fishes are a prominent representative of the region's ichthyofauna, highlighting its unique biodiversity (Wu and Tan, 1991). Xizang, located on the southwestern frontier of China, is not only the heartland of the Qinghai-Tibet Plateau but also one of the highest regions in the world. Here, schizothoracin fishes demonstrate significant uniqueness in terms of species composition, geographical distribution, and ecological status. The Yarlung Zangbo River winds through the southern part of Xizang, flowing from west to east, and is home to a rich diversity of animal, plant, and microbiota life. According to the Tibet Autonomous Region Fisheries Bureau in 1995, the river system is host to four genera and nine species of schizothoracin fishes. In its middle reaches, endemic species such as *Oxygymnocypris stewarti, Schizothorax waltoni, Schizothorax macropogon, Ptychobarbus dipogon, Schizothorax o'connori* Lloyd, and *Schizopygopsis younghusbandi* can be found. These species are not only unique to the Yarlung Zangbo River but also constitute important indigenous economic fish. They hold significant value in fisheries production and the development and utilization of resources (Tibet Autonomous Region Fisheries Bureau, 1995; Zhao et al., 2008; Yang et al., 2010). Moreover, *O. stewarti, S. waltoni* and *S. macropogon* are national second-class protected animals in China (State Forestry and Grass Industry Bureau, 2021). *O. stewarti* and *S. macropogon* are endangered fish (Jiang et al., 2016). In recent years, the schizothoracin fishes in the Yarlung Zangbo River have been increasingly affected by human activities and environmental changes, leading to a noticeable decline in fisheries resources in certain areas. The schizothoracin fishes of Xizang exhibit biological characteristics such as slow growth rate, long lifespan, late sexual maturity, and low fecundity, rendering their genetic resources extremely fragile. Once damaged, the likelihood of recovery is minimal (Tibet Autonomous Region Fisheries Bureau, 1995). Studies have found that the composition and diversity of intestinal microbiota in cold-water fish with different diets in the upper reaches of the Yangtze River vary, affecting nutrient absorption (Xu et al., 2022). The intestinal tract microorganisms of wild fish in the Wuhan section of the Yangtze River mostly originate from the environment and are related to diet, similar to the sarcophagy fish communities in the same habitat (Yang et al., 2022). It implies that the microbiota of cold-water fish at high altitudes is affected by similar factors. Meanwhile studies have shown that captivity will change the intestinal microbiota of animals (West et al., 2019). Therefore, it is important to understand the intestinal microorganisms of fish caught in the wild in the protection of endangered fish.

High-throughput sequencing technology has emerged as a cutting-edge method for studying gut microbiota, enabling the analysis of microbiota community structure and diversity through sequencing depth and phylogenetic relationships (Xiang et al., 2022). For instance, Xiao and colleagues used this technology to analyze the gut microbiota of three fish species—*Culter alburnus*, C. *dabryi*, and *Cultrichthys erythropterus*—in QianXia Lake, Lishui City, Zhejiang, revealing similar microbiota structures (Xiao et al., 2022). Additionally, Cui and others employed high-throughput sequencing to explore the gut microbiota community characteristics of wild *Grammoplites scaber* in the Pearl River Estuary. Their findings indicated the coexistence of probiotics and pathogens in the gut, with a trend toward functional antagonism (Cui et al., 2022). Currently, high-throughput sequencing technology has been extensively used in China for researching the gut microbiota of various freshwater fish, including phytophagous, filter-feeding, and sarcophagy species (Wang et al., 2014; Liu et al., 2016; Li et al., 2016; Xiao et al., 2022). No studies have yet applied this technique to fish species endemic to Xizang. However, explorations have been carried out on the adaptability of microorganisms to fish in high-altitude and cold-water environments, such as Zhao et al. revealed through metagenomic methods that microorganisms such as Proteobacteria were dominant in the high-altitude salt lakes of the Qinghai-Tibet Plateau (Zhao et al., 2024); Tong et al. analyzed the high-altitude adaptability of naked carp in Qinghai Lake through transcriptomics, and the changes in their gene expression might be associated with the intestinal microbiota (Tong et al., 2017). These studies have provided important reference basis at the environmental and physiological levels for the research on the microbiota of fish endemic to Xizang.

Based on the known evidence of the association between the vertebrate microbiome and host adaptability (Huang et al., 2022; Cox et al., 2022), this study verified the following three core hypotheses: ① Compared with least concern species, the diversity of the gut microbiota of endangered Xizang fish was significantly reduced; ② Dietary type is the primary factor driving the diversity of microbiota in different fish species; ③ The endangered status is associated with specific microbial markers.

## 2 Materials and methods

### 2.1 Overview of the study area

**Figure 1:**
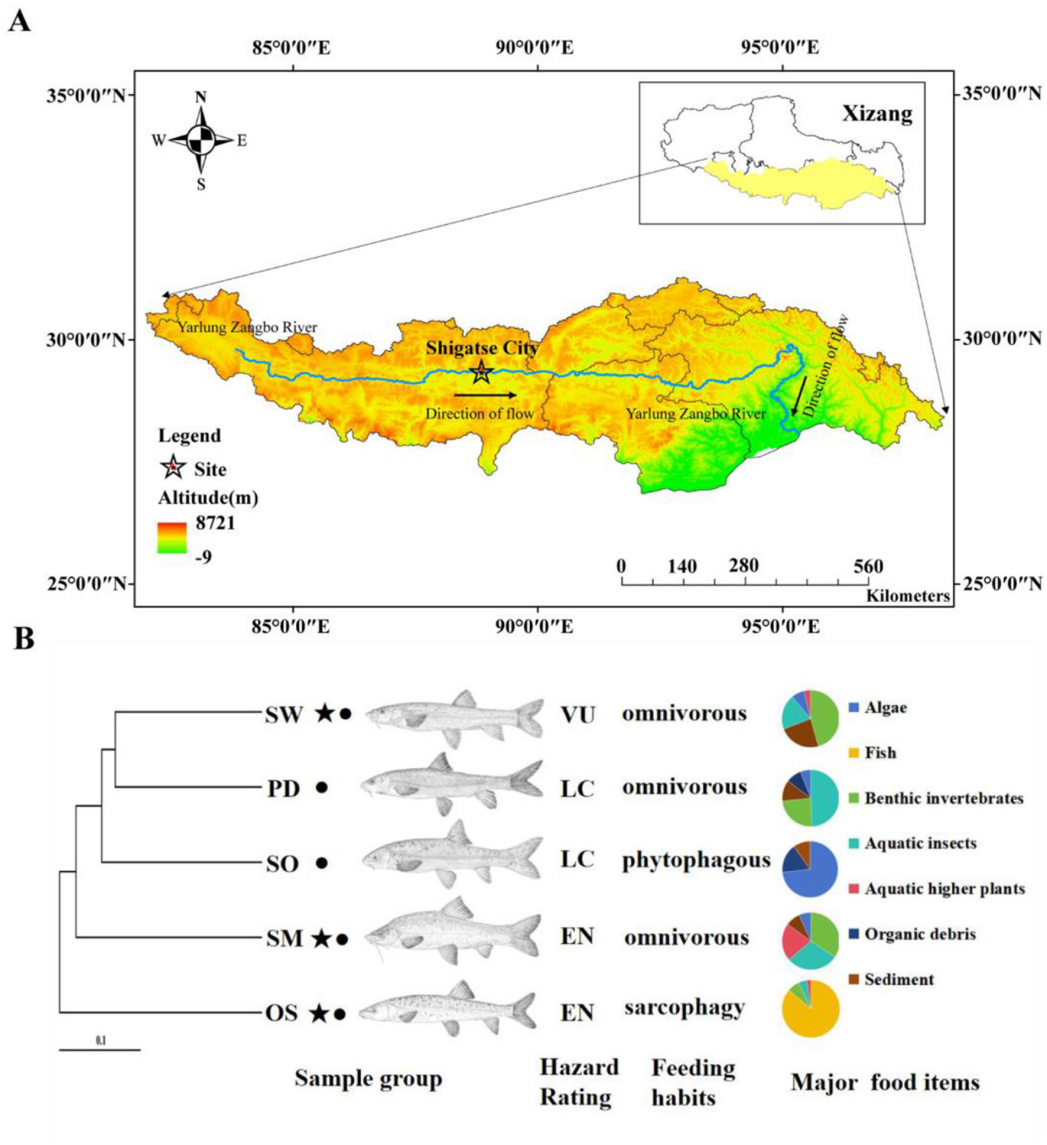
**(A)** Sample collection sites. **(B)** Host phylogeny and sample collection information. OS, *Oxygymnocypris stewarti;* SO, *Schizothorax o ' connori* Lloyd; PD, *Ptychobarbus dipogon*; SW, *Schizothorax waltoni*; SM, *Schizothorax macropogon*. ⋆, the national second-class protected animals in China; •, the fish endemic to Xizang. LC, Least Concern; VU, Vulnerable; EN, Endangered. Major food items data sourced from Ji (2008).

The Yarlung Zangbo River (Figure 1A) is the highest altitude river in China, originating from the Jemayangzong Glacier. It flows from west to east across the southern part of the Qinghai-Tibet Plateau, and after circumnavigating Mount Namcha Barwa, it heads south into the Indian Ocean. Within the territory of Xizang, the river stretches for 2,057 kilometers with a basin area of about 935,000 square kilometers. The middle reaches of the Yarlung Zangbo extend from Zhongba County to Milin County, covering a river length of approximately 1,300 kilometers, at an altitude above 3,000 meters (Guan et al., 1984). This region includes areas like the middle and lower reaches of the Lhasa River and Nyang River, with an annual average precipitation ranging between 300 and 600 millimeters.

### 2.2 Sample collections

In the summer of 2017, the experimental fish samples were collected by trawling in the middle reaches of Yarlung Zangbo River in Lhatse County, Shigatse (Figure 1A). Longitude 87°22 “east longitude, dimension 28°44” north latitude. In total, we selected fish species with different feeding habits, including six of *O. stewarti*; omnivorous diet *S. waltoni* has six, *S. macropogon* has seven, *P. dipogon* has six; and five of *S. o'connori* Lloyd, which primarily consume phytoplankton (Figure 1B). All sampled fish displayed normal external appearance and showed no apparent signs of disease or illness.

During the collection of fish specimens, fish were anesthetized using a 50 mg/L anesthetic solution. Subsequently, the fish's body surface was wiped with 75% alcohol, and under sterile conditions, the abdominal cavity was carefully incised. Bloodstains on the abdominal cavity surface were absorbed using absorbent paper. The external wall of the intestine was rinsed multiple times with 0.9% sterile physiological saline. The entire intestinal tract of the fish was removed, and the anterior and middle sections of the intestine were carefully excised using scissors. Specifically, we selected intestinal segments with a luminal content stage of two phase or higher. Immediately after sampling, the samples were stored in a sterile centrifuge tube for flash freezing in liquid nitrogen, and then transported back to the laboratory and stored in an ultra-low temperature refrigerator at −80°C until the samples were sent for sequencing.

### 2.3 16S rRNA sequencing

We used the CTAB method to extract genomic DNA from the samples. Subsequently, we assessed the purity and concentration of the DNA using agarose gel electrophoresis. An appropriate amount of sample DNA was then transferred to a centrifuge tube, and we diluted it to a concentration of 1 ng/μL using sterile water. Using the diluted genomic DNA as a template, we targeted the 16S V4 region and performed PCR amplification. The PCR reaction utilized the Phusion^®^ High-Fidelity PCR Master Mix with GC Buffer from New England Biolabs and a high-fidelity enzyme. The primers used for the 16S V4 region were 515F (5′-GTGCCAGCMGCCGCGGTAA-3′; Parada et al., 2014) and 806R (5′-GGACTACHVGGGTWTCTAAT-3′; Apprill et al., 2015). The PCR products were subjected to electrophoresis on a 2% agarose gel to assess their concentration. Following this assessment, an equal amount of PCR product was mixed and thoroughly combined. The mixed product was then purified using a 2% agarose gel with 1 × TAE buffer to cut and recover the target bands. Subsequently, library construction was carried out using the Ion Plus Fragment Library Kit 48 rxns from Thermofisher. After successful library construction, the prepared library underwent quantification using Qubit and passed library quality checks. Finally, sequencing was performed using the Thermofisher Ion S5 TMXL platform.

### 2.4 Sequence data processing

We initially processed the reads using the Cutadapt software (Martin, 2011). This involved trimming the low-quality portions of the reads and subsequently splitting the obtained reads into individual sample data based on barcodes. We then removed the barcode and primer sequences to obtain raw reads. Following this preprocessing, the reads underwent removal of chimeric sequences. The reads were aligned with a species annotation database (Edgar et al., 2011) to detect chimeric sequences, and these chimeric sequences (Haas et al., 2011) were ultimately eliminated to obtain high-quality sequences. Subsequently, we performed OTU clustering and species classification analysis at a 97% similarity threshold using Uparse v7.0.1001 (Edgar, 2013). Based on the results of OTU clustering, species annotations were assigned to representative sequences of each OTU. Mothur software was used to perform species annotation analysis against the SILVA (Wang et al., 2007) SSUrRNA database (Quast et al., 2013) using the Mothur method (threshold set between 0.8 and 1), resulting in species information corresponding to the OTUs. For multiple sequence alignment, we used the MUSCLE software (Version 3.8.31; Edgar, 2004). This allowed us to establish the phylogenetic relationships among all OTU representative sequences. Qiime 1.9.1 was employed to detect shared and unique OTUs across different sample groups. Subsequently, data normalization was performed on all samples. The normalization was based on the sample with the lowest sequence count SW.1, which had 48,964 sequences. All subsequent alpha diversity and beta diversity analyses were conducted using the normalized data.

### 2.5 Statistical analysis

Alpha diversity analysis was conducted using Qiime software (Version 1.9.1) to calculate Chao1, Shannon, and Simpson indices. The plots were generated using Rstudio 4.3.0. Rarefaction curves, Rank abundance curves, and other visualizations were created using R software (Version 2.15.3). Beta diversity analysis was also performed using Qiime software (Version 1.9.1). The PCoA and NMDS plots were plotted using Rstudio 4.3.0 based on the Bray-Curtis distance, weighted and Unifrac distances. The differences between the Alpha and Beta diversity groups were analyzed using the Tukey test and the wilcox test of the agricolae package. Finally, the results of each index data were analyzed using the data obtained after the wilcox test. LEfSe analysis was performed using Galaxy software, and default settings with an LDA Score threshold of 4 were used for visualization. Inter-group differences were assessed using Anosim analysis, calculated using the anosim function from the vegan package in R. Significant species differences among groups were determined through inter-group *T*-test analysis in R and the results were visualized. Other software used in this study included Excel 2021, ArcMap, and Adobe Illustrator 2023.

## 3 Results

### 3.1 Overview of sequencing data results

**Figure 2:**
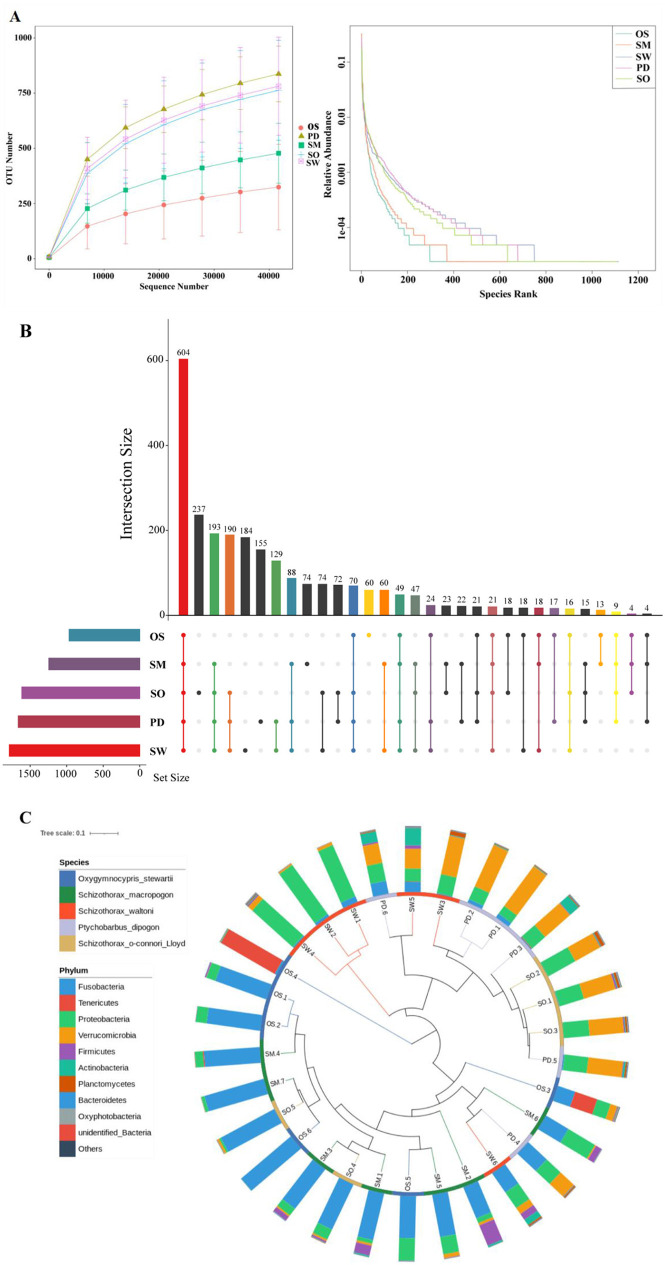
**(A)** Species Sparse Curve. **(B)** OTU Upset diagram of five fish species unique or shared. **(C)** Gate-level grouping of microbiota diversity by host phylogeny and host data. The inner circle branches are colored by host type (blue = *O. stewarti*; green = *S. macropogon*; red = *S. waltoni*; purple = *P. dipogon;* brown = *S. o'connori* Lloyd). The outer ring is the relative abundance of the phylum of microorganisms in each host.

The statistical analysis and processing of sequencing results were conducted, including the calculation of sequencing read counts, data yield, and the sequencing error rate for the final sequences (Appendices Table A1). The total number of raw sequences obtained from all sample groups was 2,399,741 reads. After quality control and filtering, there were a total of 2,294,817 sequences. The species rarefaction curve reached a plateau, indicating that the sequencing depth adequately represented the predominant microbiota communities in the samples (Figure 2A). At the OTU level, a total of 604 OTUs were shared among all sample groups. Additionally, there were 60 unique OTUs in the *O. stewarti* group, 74 unique OTUs in the *S. macropogon* group, 184 unique OTUs in the *S. waltoni* group, 155 unique OTUs in the *P. dipogon* group, and 237 unique OTUs in the *S. o'connori* Lloyd group (Figure 2B). Reflecting the relative abundance of microorganisms at the phylum level to fish hosts explains some clustering of microbial composition by the hosts (Figure 2C). While similar species abundances were generally observed within the same fish groups, some differences were evident, such as a distinct branch for the *O. stewarti* group and clustering with the *S*. *macropogon* group.

### 3.2 Composition and structure of intestinal flora of five fish species

**Figure 3:**
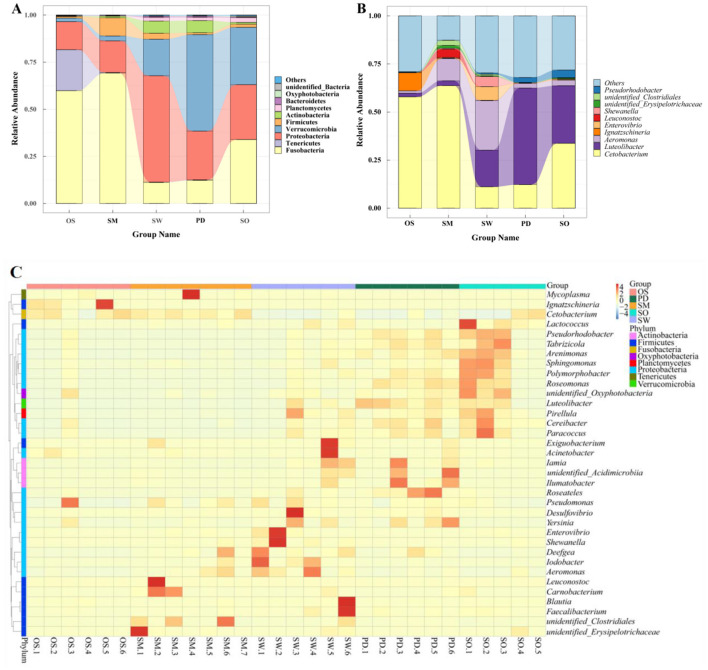
Different levels of gut microbiota in five fish, Phylum **(A)**, Genus **(B)**, **(C)** Cluster heat map of species abundance of 35 genera.

Fusobacteria, Proteobacteria, and Verrucomicrobia are the three major dominant phyla among the gut bacteria of the five fish species (Figure 3A). Among them, Fusobacteria and Proteobacteria are predominant in all groups, while the *O. stewarti* group has the unique dominant phylum Tenericutes (21.76%). The *S. macropogon* group is primarily composed of Fusobacteria (69.23%), Proteobacteria (16.80%), and Firmicutes (9.61%) as dominant phyla. The *S. waltoni* group is mainly characterized by Proteobacteria (56.47%), Verrucomicrobia (19.39%), and Fusobacteria (11.16%) as dominant phyla. The *P. dipogon* group is primarily composed of Verrucomicrobia (51.22%), Proteobacteria (25.88%), and Fusobacteria (12.30%) as dominant phyla. The *S. o'connori* Lloyd group is mainly characterized by Fusobacteria (33.93%), Verrucomicrobia (30.44%), and Proteobacteria (29.09%) as dominant phyla. The bacterial community composition of the *S. waltoni* and *P. dipogon* groups at the phylum level is the most similar (Figure 3A).

At the genus level, each group exhibits common dominant genera as well as unique genera, forming their distinctive gut microbiota community structures. As shown in Figure 3B: The dominant genera in the *O. stewarti* group are *Cetobacterium* (57.89%) and *Ignatzschineria* (9.38%), and a few *Aeromonas* (1.24%) and *Luteolibacter* (1.90%). The dominant genera in the *S. macropogon* group are *Cetobacterium* (63.72%), *Aeromonas* (11.58%) and *Leuconostoc* (4.31%), followed by unidentified *Clostridials* (2.65%), Luteolibacter (2.53%) and unidentified- Erysipolitrichaceae (1.45%). The dominant genera in the *S. waltoni* group are *Aeromonas* (25.78%), *Luteolibacter* (19.05%), *Cetobacterium* (11.13%), *Enterovibrio* (7.08%) and *Shewanella* (5.27%). The dominant genera in the *P. dipogon* group are *Luteolibacter* (50.19%), *Cetobacterium* (12.28%), *Pseudorhodobacter* (2.62%) and *Aeromonas* (2.44%). The dominant genera in the *S. o'connori* Lloyd group are *Cetobacterium* (33.73%), *Luteolibacter* (29.98%), *Pseudorhodobacter* (3.97%) and *Aeromonas* (2.82%). Additionally, a significant number of other genera were detected in each species, accounting for 12.62% to 32.03% of the total abundance in each group, with the order of abundance being *S. macropogon*<*O. stewarti*<*S. waltoni*<*S. o'connori* Lloyd < *P. dipogon*. The community structures can be roughly categorized as *O. stewarti* being similar to *S. macropogon, S. macropogon* being similar to *S. waltoni*, and *P. dipogon* being similar to *S. o'connori* Lloyd. Clustering of representative bacteria from the top 35 genera (Figure 3C) revealed significant differences both between and within species.

### 3.3 Gut microbiota diversity of five fish species

**Figure 4:**
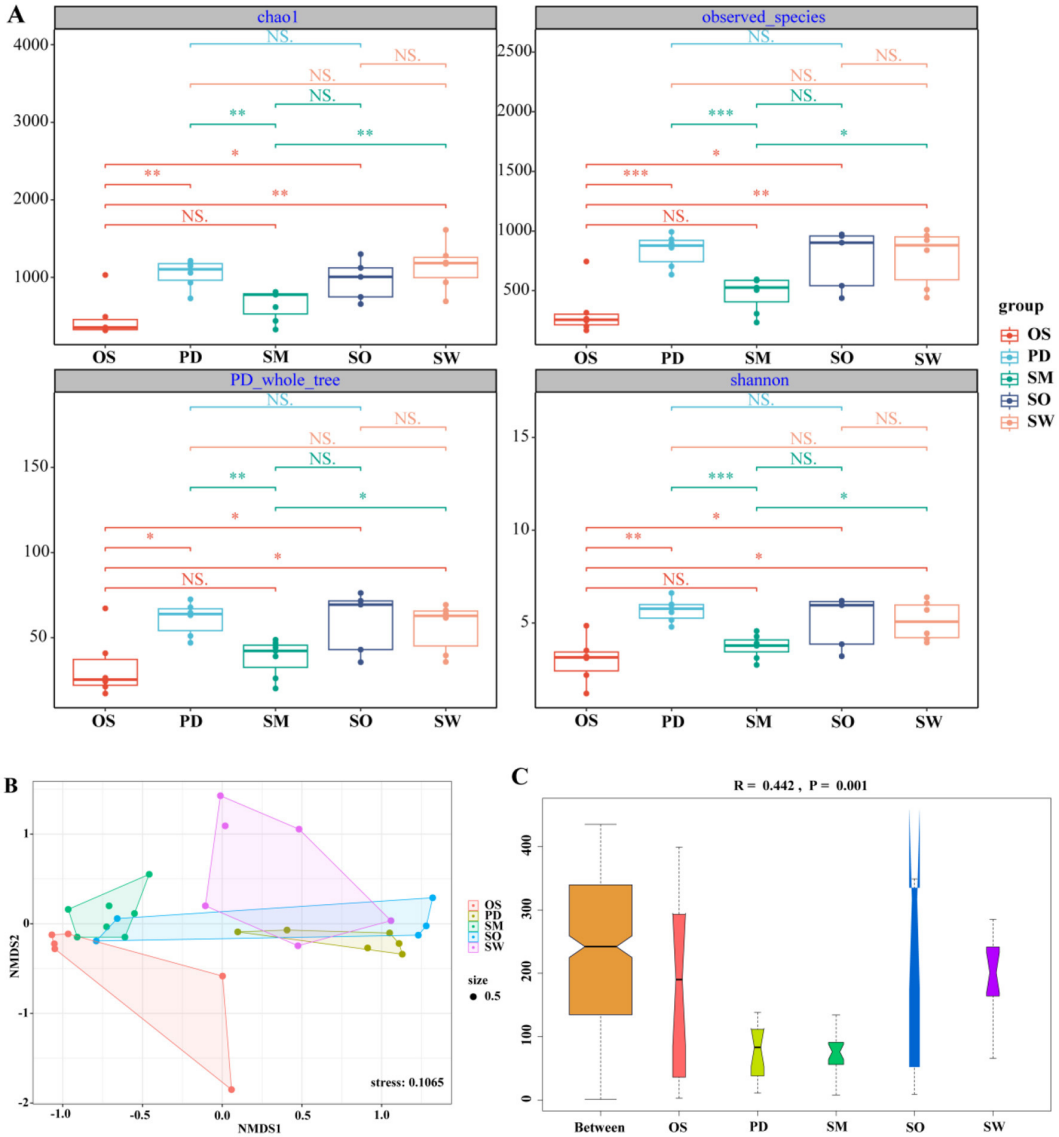
**(A)** Box plot of Chao1, shannon, observed_species, PD_whole_tree index component difference. The center square is the mean, the center line is the median, the box outline is equal to 1 standard deviation, the colored dots are the sample, *** indicates *P* < 0.001, showing an extremely significant difference; ** indicates *P* < 0.01, showing an extremely significant difference; * indicates *P* < 0.05, showing a significant difference; NS indicates *P* > 0.05, indicating no significant difference. **(B)** NMDS diagram of gut microbiota based on Bray-Curtis distance. When stress<0.2 can be represented by the two-dimensional dot diagram of NMDS, which has certain explanatory significance. **(C)** Anosim group difference diagram. R > 0, indicating that the difference between groups was greater than the difference within the group, indicating that there was a difference between the groups, and *P* < 0.05, indicating a significant difference between the groups.

At the OTU level, the α-diversity analysis of gut microbiota communities among groups, including Chao1, observed_species, PD_whole_tree, and Shannon index, showed that the α-diversity indices were highest in the *P. dipogon* group (Appendices Table A2). From the perspective of species richness in microbiota communities, the order of species richness among groups was *P. dipogon* > *S. waltoni* > *S. o'connori* Lloyd > *S. macropogon* > *O. stewarti*. Specifically, *O. stewarti* exhibited significant differences compared to *P. dipogon, S. o'connori* Lloyd, and *S. waltoni*, while *S. macropogon* showed significant differences compared to *S. waltoni*, and *P. dipogon* showed significant differences compared to *S. macropogon*. This pattern reflects the diversity levels of microbiota communities among the groups (Figure 4A).

To assess the similarity or dissimilarity of gut microbiota communities among groups, non-metric multidimensional scaling analysis (NMDS) based on Bray–Curtis distance was conducted to visualize the data reduction (stress = 0.1065; Figure 4B). The NMDS plot shows that the *O. stewarti* group forms a distinct cluster and is clearly separated from the *P. dipogon* and *S. waltoni* groups, indicating that its gut microbiota community is influenced by nutritional factors. However, there is some degree of overlap between *P. dipogon, S. o'connori* Lloyd, and *S. waltoni*, suggesting that they share some similarities in addition to their differences. This also suggests the presence of other factors influencing their gut microbiota communities, or the need for more detailed analysis. ANOSIM analysis (Figure 4C) also indicates that there is a significant difference in the composition of gut microbiota communities among the five fish species (*R* = 0.442, *P* = 0.001).

### 3.4 Biomarkers among five fish species

**Figure 5:**
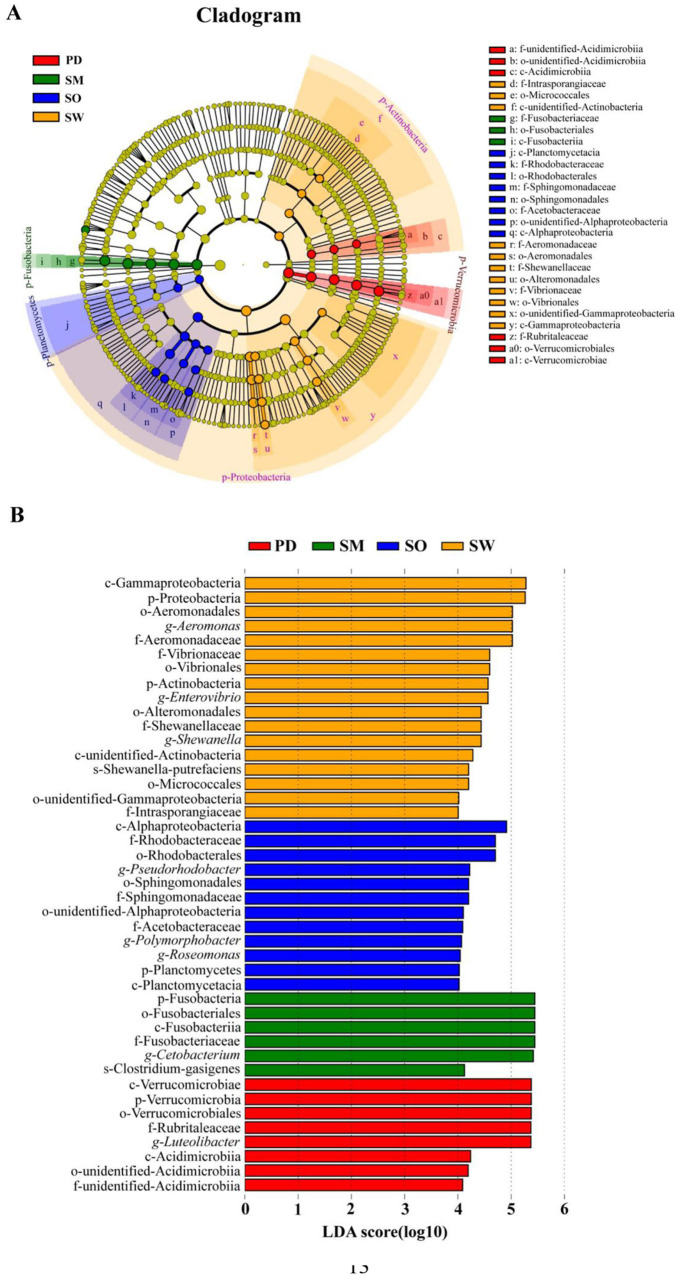
LEfSe analysis of gut microbiota in five fish. **(A)** Evolutionary clade diagram, **(B)** Histogram of the distribution of different species. Among them, LDA value ≥4.

The LEfSe analysis results revealed a total of 5 phyla, 7 classes, 11 orders, 10 families, 8 genera, and 2 species of significant species with relative abundances that differed significantly among different fish species (Figure 5A). The *S.waltoni* group had the highest number of differentially abundant taxonomic units, followed by *S. o'connori* Lloyd and *P. dipogon*, while *S. macropogon* had the fewest, and *O. stewarti* had no differentially abundant species in this grouping (Figure 5B). The most abundant differentially abundant species in the *S. waltoni* group included Gammaproteobacteria, Proteobacteria, Aeromonadales, *Aeromonas*, and Aeromonadaceae; in the *S. o'connori* Lloyd group, it included Alphaproteobacteria, Rubritaleaceae, and Rhodobacterales; in the *P. dipogon* group, it included Verrucomicrobiae, Verrucomicrobia, Verrucomicrobiales, Rubritaleaceae, and *Luteolibacter*; and in the *S. macropogon* group, it included Fusobacteria, Fusobacteriales, Fusobacteriia, Fusobacteriaceae, and *Cetobacterium*.

### 3.5 Comparison of gut microbiota in fish among different dietary preferences

After considering various influencing factors, *O. stewarti, S. waltoni, S. macropogon, P. dipogon*, and *S. o'connori* Lloyd—five species of highland schizothoracine fishes—were classified into three dietary categories based on existing literature (Ji, 2008): *O. stewarti* belongs to sarcophagy (SAR); *S. waltoni, S. macropogon* and *P. dipogon* belong to omnivorous (OMN); and *S. o'connori* Lloyd belong to phytophagous (PHY; Figure 1B). This classification facilitates the comparison of gut microbiota community structures among highland schizothoracine fishes with different dietary preferences.

**Figure 6:**
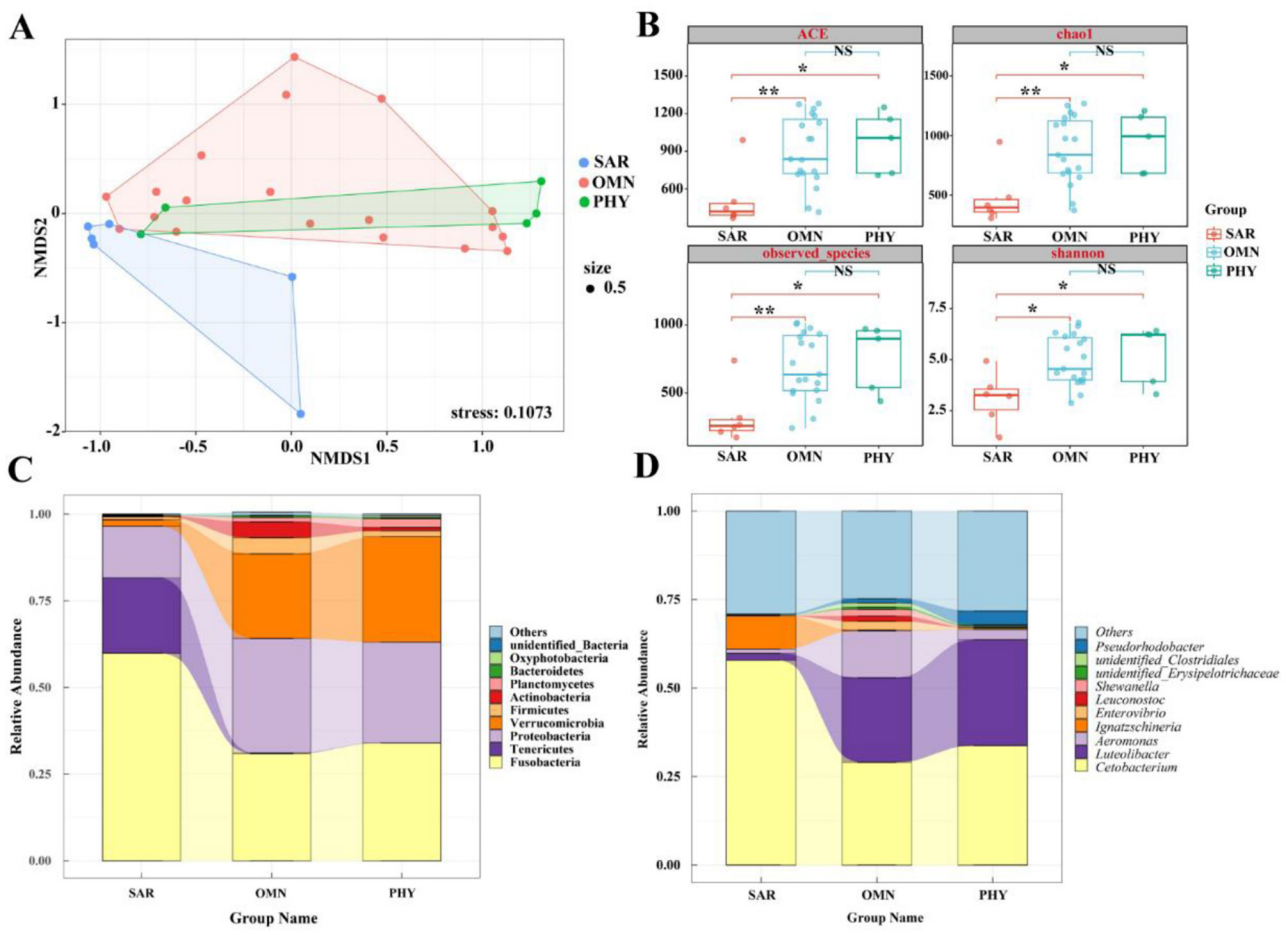
**(A)** NMDS diagram of gut microbiota based on Bray-Curtis distance. When stress<0.2 can be represented by the two-dimensional dot diagram of NMDS, which has certain explanatory significance. **(B)** Box diagram of differences between groups of Alpha diversity index with different feeding habits. The center square is the mean, the center line is the median, the box outline is equal to 1 standard deviation, the colored dots are the sample, ** indicates *P* < 0.01, showing an extremely significant difference; * indicates *P* < 0.05, showing a significant difference; NS indicates *P* > 0.05, indicating no significant difference. **(C)** Top 10 with different feeding habits at the phylum level. **(D)** Top 10 with different feeding habits at genus level.

To compare the similarity or differences in microbiota community structures among groups with different dietary preferences, non-metric multidimensional scaling analysis (NMDS) was employed to reduce the dimensionality of the data for visualization. Figure 6A demonstrates that sarcophagy fish are significantly separated from omnivorous and phytophagous fish, indicating larger differences. While there is some overlap between omnivorous and phytophagous fish. This suggests that, in addition to the differences, there are also certain similarities. At the level of Alpha diversity, four diversity indices were selected: ACE, Chao1, observed_species, and Shannon. Figure 6B shows that the ACE, Chao1, and observed_species indices for sarcophagy fish significantly differ from those for omnivorous fish (^**^P < 0.01) and show a significant difference when compared to phytophagous fish (^*^P < 0.05). There is no significant difference between the omnivorous group and the phytophagous group (*P* > 0.05).

At the phylum level, Figure 6C shows that the gut microbiota of sarcophagy fish is primarily composed of Fusobacteria (59.86%), Tenericutes (21.76%), Proteobacteria (14.85%), and Verrucomicrobia (1.94%). Omnivorous fish are mainly composed of Proteobacteria (33.05%), Fusobacteria (30.90%), Verrucomicrobia (24.41%), Actinobacteria (4.61%), and Planctomycetes (1.24%). Phytophagous fish, on the other hand, are primarily composed of Fusobacteria (33.93%), Verrucomicrobia (30.44%), and Proteobacteria (29.09%), followed by Planctomycetes (2.48%) and Firmicutes (1.70%). It can be intuitively seen from Figure 6C that the microbiota compositions of omnivorous and phytophagous fish are the most similar.

At the genus level, Figure 6D shows that the gut microbiota of sarcophagy fish is dominated by *Cetobacterium* (57.89%), followed by *Ignatzschineria* (9.38%), other genera (29.05%) and *Aeromonas* (1.24%). The microbiota of omnivorous fish is mainly composed of *Cetobacterium* (29.04%), other genera (24.78%), *Aeromonas* (13.26%), *Luteolibacter* (23.92%), *Enterovibrio* (2.48%), *Shewanella* (1.93%), *Leuconostoc* (1.45%) and *Pseudorhodobacter* (1.13%). The gut microbiota of phytophagous fish primarily consisting of *Cetobacterium* (33.73%), *Luteolibacter* (29.98%) and other genera (28.18%), followed by *Pseudorhodobacter* (3.97%) and *Aeromonas* (1.24%). From Figure 6D, it can be seen that the difference of dominant bacteria in fish with different feeding habits mainly lies in different contents. The species of intestinal bacteria in phytophagous fish and omnivorous fish are similar, *Luteolibacter* occupies a certain proportion, while the dominant species of intestinal flora difference in sarcophagy fish is *Ignatzschineria*.

### 3.6 Comparison of gut microbiota in fish among different conservation statuses

Among the five species of highland schizothoracine fishes, three are classified as second-class protected animals in China. The species *O. stewarti, S. waltoni, S. macropogon, P. dipogon*, and *S. o'connori* Lloyd were categorized into conservation statuses according to the According to the Red List of Vertebrates in China and the Red List of China of Threatened Species (Wang and Xie, 2009; Jiang et al., 2016). These species were divided into three groups: Endangered (EN), Vulnerable (VU), and Least Concern (LC; Figure 1B). The endangered category includes *O. stewarti* and *S. macropogon*, the vulnerable category includes *S. waltoni*, and the least concern category includes *P. dipogon* and *S. o'connori* Lloyd. A comparison of the gut microbiota community structures of highland schizothoracine fishes across different conservation statuses was conducted.

**Figure 7:**
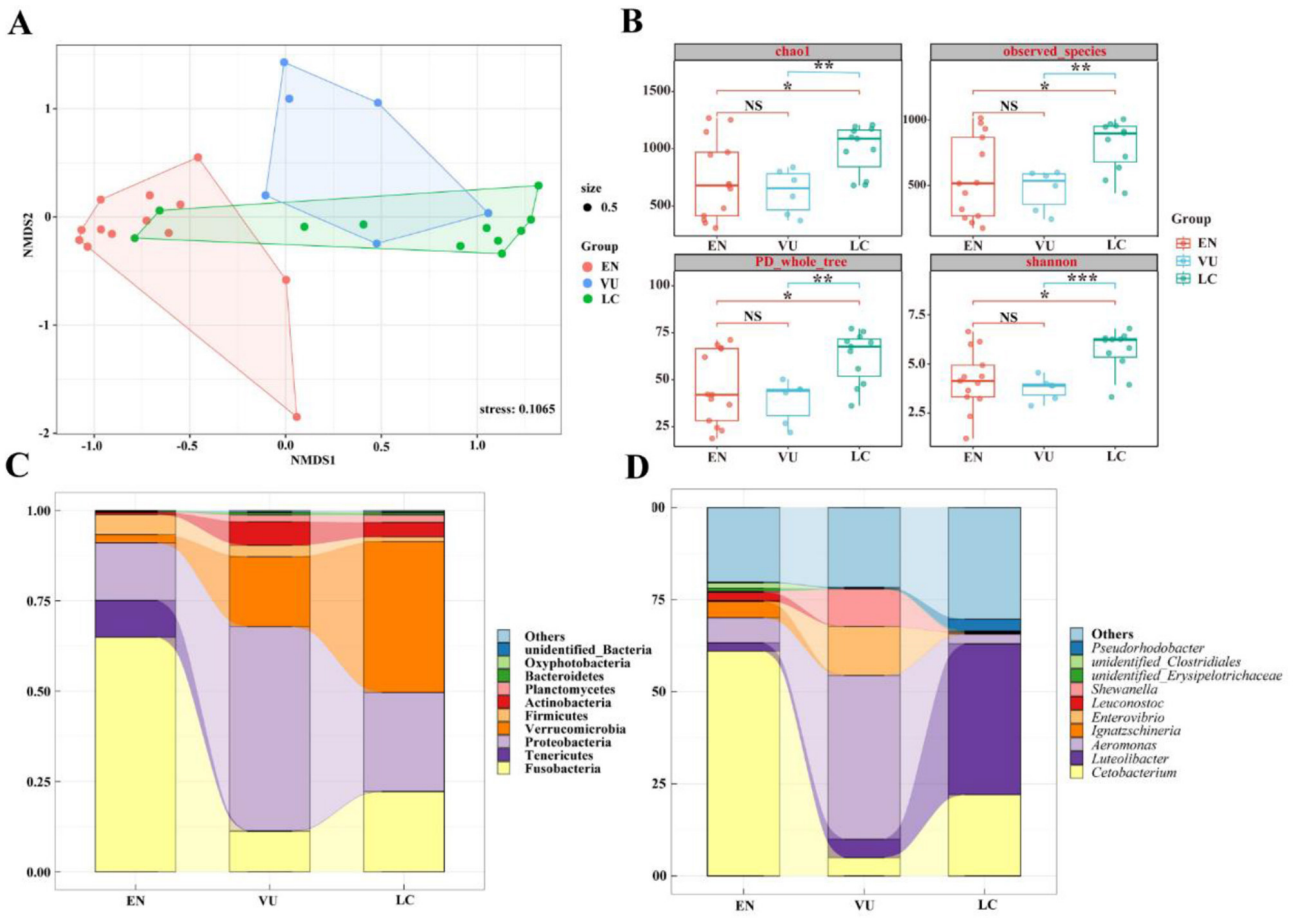
**(A)** NMDS diagram of gut microbiota based on Bray-Curtis distance. When stress < 0.2 can be represented by the two-dimensional dot diagram of NMDS, which has certain explanatory significance. **(B)** Box diagram of differences between groups of Alpha diversity index. The center square is the mean, the center line is the median, the box outline is equal to 1 standard deviation, the colored dots are the sample, *** indicates *P* < 0.001, showing an extremely significant difference; ** indicates *P* < 0.01, showing an extremely significant difference; * indicates *P* < 0.05, showing a significant difference; NS indicates *P* > 0.05, indicating no significant difference. **(C)** Top 10 at the phylum level. **(D)** Top 10 at genus level.

To compare the similarity or differences in microbiota community structures among groups with different conservation statuses, non-metric multidimensional scaling analysis (NMDS) was used to reduce the dimensionality of the data for visualization. Among different conservation statuses, Figure 7A shows that the endangered group, the vulnerable group, and the least concern group can all be significantly separated. Fish in the endangered group form a distinct cluster, showing relatively larger differences from the other two groups, while the vulnerable group and the least concern group have some overlapping clusters. As shown in Figure 7B, at the Alpha diversity level, four diversity indices were selected: Chao1, observed_species, PD_whole_tree, and Shannon. The vulnerable group's ACE, Chao1, and observed_species indices differ significantly from those of the least concern group (*P* < 0.001, *P* < 0.01), with no significant difference from the endangered group (*P* > 0.05). The endangered group shows significant differences from the least concern group (*P* < 0.05).

Figure 7C presents the top 10 species in terms of relative abundance at the phylum level, showing that the main dominant phyla are consistent across groups, but with significant differences in content. For instance, the endangered group is composed of Fusobacteria (64.90%), Proteobacteria (15.90%), Tenericutes (10.19%), Firmicutes (5.56%), and Verrucomicrobia (2.31%); the vulnerable group consists of Proteobacteria (56.47%), Verrucomicrobia (19.39%), Fusobacteria (11.16%), Actinobacteria (6.48%), Firmicutes (3.17%), and Planctomycetes (1.87%); and the least concern group primarily includes Verrucomicrobia (41.77%), Proteobacteria (27.34%), Fusobacteria (22.13%), Actinobacteria (3.9%), Planctomycetes (2.05%), and Firmicutes (1.31%).

As shown in Figure 7D, which displays the top 10 species in terms of relative abundance at the genus level, there are considerable differences in dominant species among the groups. The endangered group is predominantly occupied by *Cetobacterium* (61.03%) and other genera (20.20%), followed by *Aeromonas* (6.81%), *Ignatzschineria* (4.44%), and *Luteolibacter* (2.24%). In the vulnerable group, *Aeromonas* (44.40%), *Enterovibrio* (13.27%), *Shewanella* (10.15%), and other genera (21.70%) have higher proportions, followed by *Luteolibacter* (5.01%) and *Cetobacterium* (4.96%). The least concern group is mainly composed of *Luteolibacter* (41.01%), other genera (30.28%), and *Cetobacterium* (22.03%), with *Pseudorhodobacter* accounting for 3.23% and *Aeromonas* for 2.61%. From Figures 6C, D, it can be seen that the species richness of gut microbiota of fish in least concern (LC) group is the highest, followed by that in vulnerable (VU) group, while that in endangered (EN) group is the lowest.

## 4 Discussion

Numerous studies have consistently shown that the composition of fish gut microbiota is complex and includes Proteobacteria, Fusobacteria, Firmicutes, *Bacteroidetes*, Actinobacteria, Chloroflexi, and Verrucomicrobia (Xing, 2013; Liu et al., 2017; Llewellyn et al., 2014). Among these, Fusobacteria can colonize the digestive tract of fish and are relatively abundant in some fish species, while Proteobacteria are the most common and abundant microbes in the fish gut, constituting more than 50% of the total gut microbiota in many fish species (Roeselers et al., 2011; Sullam et al., 2012; Xia et al., 2014). In this study, Fusobacteria, Proteobacteria and Verrucomicrobia are the dominant bacteria in all groups, regardless of species, feeding habits or endangered levels, but their abundance differences are significant, indicating that the gut microbiota is the result of species-specific selection. In addition, Tenericutes as a unique dominant species in the endangered (EN) group, is a unique group of microbiota that are difficult to cultivate. According to the results of metagenome prediction, Tenericutes has outstanding nucleic acid degradation ability, which plays an important role in driving the cycle of phosphorus, nitrogen and other elements, and has unique research value in evolution, pressure resistance and element cycle (Zheng et al., 2023). It was found that the intestinal microbes of honey bees cooperated with the host to metabolze phytotoxins, which revealed that microbes could assist the host to cope with environmental stress (Motta et al., 2022). Demonstrated that metabolites of microbiota can interfere with mitochondrial energy metabolism of host cells, inhibit pathogens, and enhance host resistance to infection (Funkhouser-Jones et al., 2023). Our results suggested that Tenericutes might help endangered fish to resist survival challenges through similar mechanisms of synergistic metabolism, energy regulation and immunity. Proteobacteria (56.47%), Verrucomicrobia (19.39%) and Fusobacteria (11.16%) are the main dominant bacteria of vulnerable (VU) fish. The research shows (Shin et al., 2015) that Proteobacteria, as a first-line responder, is sensitive to environmental factors (such as diet) and related to indigestion. In addition to exogenous enteropathogenic bacteria, the intestines of healthy mammals also contain several symbiotic bacteria belonging to this phylum as their natural intestinal flora (Shin et al., 2015). The main dominant bacteria of least concern (LC) fish are Verrucomicrobia (41.77%), Proteobacteria (27.34%) and Fusobacteria (22.13%). Verrucomicrobia exists in the inner layer of intestinal mucosa and exists in large quantities in healthy individuals. They can decompose polysaccharides, such as mucopolysaccharides and cellulose, so as to provide energy and nutrients.

Alpha diversity analysis indicates significant differences between *O. stewarti* and *P. dipogon, S. o'connori* Lloyd, and *S. waltoni*, as well as between *S. macropogon* and *S. waltoni*, and *P. dipogon* and *S. macropogon*. Regarding gut microbiota diversity, the order from highest to lowest diversity among the groups is *P. dipogon* > *S. waltoni* > *S. o'connori* Lloyd > *S. macropogon* > *O. stewarti*. We suspect that this may be related to many factors, such as host and eating habits. At present, more and more people around the world realize that microorganisms are regarded as an important way to protect threatened species (Cox et al., 2022; West et al., 2019). This study found that the structure of the intestinal microbiota community in fish was significantly correlated with the host's diet, which was related to Pan et al. The research results on fish in the Yellow River are consistent (Pan et al., 2023), further confirming the key role of diet in shaping the intestinal microbiota of fish. In this study, we found that the diversity of gut microbiota in threatened host species was low when comparing the endangered levels. For example, *O. stewarti, S. macropogon* and *S. waltoni* are national second-class protected animals in China, and the species are classified as endangered (EN) for *O. stewarti* and *S. macropogon*, vulnerable (VU) for *S. waltoni*, and least concern (LC) for *P. dipogon* and *S. o'connori* Lloyd. The gut microbiota diversity ranking as follows: least concern (LC) > vulnerable (VU) > endangered (EN). This is consistent with the endangered level of intestinal microorganisms in vertebrate hosts studied by Huang et al.'s (2022). Cox et al. proposed in the study of reptiles that the stability of the microbiota is a key indicator for the conservation of endangered species (Cox et al., 2022). The findings of this study in fish echo this, indicating that for both reptiles and fish, maintaining the stability of the microbial community is crucial for the host to adapt to environmental changes and survive. The beta diversity analysis indicated that the gut microbiota communities were influenced by nutritional levels, as shown by NMDS, revealing not only differences but also similarities. ANOSIM highlighted significant differences in the gut microbiota composition among the five fish species.

The gut microbiota of fish is primarily composed of *Cetobacterium, Bacteroide*s, and *Fusobacterium*. *Cetobacterium* is a core member of the gut microbiota in freshwater fish and holds a significant ecological niche. *Bacteroides* has been shown to produce digestive enzymes and has a competitive advantage in the gut during periods of relative food scarcity. Our research results show that *Bacteroides* does not dominate among the dominant genera in any of the studied fish groups (Figures 3B, 4A, B). This suggests that the fish in the Yarlung Zangbo River section are not currently experiencing food scarcity. *Cetobacterium* is a common dominant bacterium in all groups, especially in the intestines of endangered fish *O. stewarti* and *S. macropogon* share *Cetobacterium* as a dominant genus. *Cetobacterium* has been shown to have the ability to synthesize vitamin B12 in freshwater fish (Sugita et al., 1997) and assists in regulating blood glucose levels, helping fish utilize carbohydrates as a source of nutrition (Wang et al., 2021). Furthermore, *Cetobacterium* and protease-producing halophiles are enriched in sarcophagy animals, indicating that bacteria with enzymatic activity, such as *O. stewarti* and *S. macropogon*, are influenced by the nutritional status. *S. macropogon* and *S. waltoni* share the genus *Aeromonas*, which is a major group in the gastrointestinal tract of freshwater fish. They release a large amount of proteases to aid in digestion. Typically, *Aeromonas* can grow in various types of food residues and establish ecological niches in the gut. They have cellulose-degrading capabilities in the gut of healthy fish but can lead to enteritis under abnormal conditions. The research results also suggest that the gut microbiota of *S. macropogon* and *S. waltoni* is similar to that of *Carassius auratus* and *Allogynogenetic crucian carp*, which are primarily omnivorous, in a study by Li et al. (2011). *Luteolibacter* is a dominant genus shared by *P. dipogon* and *S. o'connori* Lloyd, with a relatively high abundance. *Luteolibacter* is relatively common in the gut of phytophagous fish and can degrade cellulose and carbohydrates, which is related to its ecological and nutritional strategies. In this study, it was found that Aeromonas and Luteolibacter were the dominant genera in the intestinal microbiota of fish. This is consistent with the conclusion put forward by Nayak SK et al. regarding the common colonizing bacteria in freshwater and marine fish (Nayak, 2010), further verifying the common characteristics and stability of the composition of the intestinal microbiota in fish. *Ignatzschineria* is a unique dominant genus of endangered fish, but there is relatively little research on it at present. *Ignatzschineria* is mainly related to insects, especially flies, which are usually in contact with rotten substances and carrion in nature. Therefore, *Ignatzschineria* has become the dominant species in endangered fish, which we guess may be due to the contact between endangered fish and carrion. However, the composition of gut microbiota is influenced by many factors, including the diet and environment conditions of fish. Therefore, *Ignatzschineria* appears in the gut of endangered fish, and the situation is not clear and needs further study. Subsequent functional verification experiments are required, such as metabolomics and gnotobiotic animal colonization experiments.

The five Xizang endemic fish species in our study share similarities in size, morphology, and feeding ecology. They inhabit the same habitat and are typically found in the same region. Fish species that inhabit the same area often face limited food resources, especially in harsh climates like the Yarlung Zangbo River, where food resources are extremely scarce. However, our research results indicate that food in this area is not in short supply. We speculate that this might be due to the different food preferences of the five fish species. For example, *O. stewarti* primarily feeds on fish (He and Chen, 2007; Li et al., 2022), and its diet has little overlap with other fish species. In contrast, *S. macropogon, S. waltoni*, and *P. dipogon* primarily feed on benthic invertebrates, aquatic insects, and algae, but their proportions differ (Ji, 2008). *S. o'connori* Lloyd primarily feeds on algae and partially overlaps with the aforementioned fish species in terms of diet, but there are significant differences in the proportions of food consumed. By comparing food proportions (Ji, 2008) with the results of our study, we can confirm that the gut microbiota of Xizang endemic fish species is related to their feeding habits. Figure 2B shows variable unique OTUs, but there has been no examination of whether these unique OTUs are biologically relevant or are contaminants. However, the differences in the numbers of unique OTUs among different groups in Figure 2B allow for a preliminary speculation that they may play a certain role in the microbial community structure. This limitation may have a certain impact on the integrity of the study. There were significant differences among the groups when comparing the gut microbiota in feeding groups. Our results show that the gut microbiota's diversity in each group is as follows: phytophagous (*S. o'connori* Lloyd) > omnivorous (*S. macropogon, S. waltoni, P. dipogon*) > sarcophagy (*O. stewarti*). This is consistent with the results of Ward et al.'s study (Ward et al., 2009), where omnivorous fish species in Antarctica had higher gut microbiota diversity than sarcophagy fish species, and that Huang et al.'s study (Huang et al., 2022), where phytophagy had higher gut microbiota diversity than omnivores and sarcophagy. These findings provides data and technical support for the breeding and protection of endemic fish in Xizang and the ecological restoration of fish in Xizang.

In this study, through a systematic investigation of the gut microbiota communities of five endemic fish species in Xizang, it is revealed that the gut microbiota composition of plateau fish exhibits significant species-specific differences and ecological adaptation characteristics. The study found that Fusobacteria, Proteobacteria, and Verrucomicrobia are the common dominant bacterial phyla. Among them, the phylum Tenericutes, which is unique to endangered fish at the EN level (with prominent nucleic acid degradation ability), and the genus *Ignatzschineria* may assist the host in coping with the stressful environment of the plateau through element cycling and metabolic synergy. This discovery provides new evidence for understanding the symbiotic evolutionary mechanism between fish and microorganisms (Walter et al., 2011). Studies on the association between diet and microbiota show that phytophagous fish are rich in *Luteolibacter* (cellulose decomposition), omnivorous fish require diverse nutrients (protein/fiber balance), and sarcophagy fish rely on *Cetobacterium* (vitamin B12 synthesis) and *Aeromonas* (protein degradation). These findings directly guide aquaculture practices—the feed for herbivorous fish should be fortified with fiber components, sarcophagy fish need to be supplemented with specific probiotics, and omnivorous fish need to optimize the nutritional ratio. Although the SILVA database has limitations in annotating certain plateau specific strains (it is recommended to verify it subsequently through NCBI-BLAST), the microbial resource bank of fish in Xizang constructed in this study has opened up a new way for the protection of aquatic organisms on the plateau. Future research should focus on: ① Comparison between wild and captive populations; ② Microbial-host co-evolution mechanism in cross-aquatic environments; ③ Pilot application of probiotic intervention, thereby comprehensively analyzing the key role of gut microorganisms in the adaptation of plateau fish to dietary, climatic and environmental changes.

## Data Availability

The data presented in the study are stored in the BioProject repository with the accession number PRJNA1270700. The data access link is: https://www.ncbi.nlm.nih.gov/sra/PRJNA1270700.
