# Supplementary material for: Gut microbiota: a new frontier in understanding and protecting endangered plateau schizothorax fish
Source: Front Microbiol. 2025 Jun 13;16:1592312. doi: 10.3389/fmicb.2025.1592312 (PMC12202599; doi:10.3389/fmicb.2025.1592312)
Supplement: Supplementary file 1 [file Data_Sheet_1.docx]

**Appendices**

Table A.1 Output Statistics of Sequencing Data

| #Sample_name | Raw_reads(#) | Clean_Reads(#) | Base(nt) | AvgLen(nt) | Q20 | GC% | Effective% |
| --- | --- | --- | --- | --- | --- | --- | --- |
| OS.1 | 82139 | 80100 | 20247742 | 252 | 84.21 | 49.02 | 97.52 |
| OS.2 | 95631 | 92823 | 23439087 | 252 | 87.69 | 49.24 | 97.06 |
| OS.3 | 83442 | 80080 | 20192714 | 252 | 88.47 | 48.95 | 95.97 |
| OS.4 | 80502 | 80143 | 20151410 | 251 | 90.6 | 43.99 | 99.55 |
| OS.5 | 81053 | 80153 | 20256276 | 252 | 86.99 | 50.12 | 98.89 |
| OS.6 | 80652 | 80262 | 20266041 | 252 | 88.00 | 48.25 | 99.52 |
| SM.1 | 87265 | 80195 | 20207861 | 251 | 81.68 | 49.77 | 91.9 |
| SM.2 | 86158 | 80200 | 20262019 | 252 | 87.08 | 50.12 | 93.08 |
| SM.3 | 83642 | 80123 | 20221695 | 252 | 86.58 | 49.44 | 95.79 |
| SM.4 | 82604 | 80164 | 20216913 | 252 | 87.63 | 48.86 | 97.05 |
| SM.5 | 51383 | 50201 | 12662527 | 252 | 83.59 | 50.32 | 97.70 |
| SM.6 | 96704 | 91215 | 22915651 | 251 | 83.09 | 51.43 | 94.32 |
| SM.7 | 82021 | 80169 | 20256055 | 252 | 83.8 | 48.59 | 97.74 |
| SW.1 | 51935 | 48964 | 12341566 | 252 | 82.76 | 53.67 | 94.28 |
| SW.2 | 85001 | 80184 | 20197060 | 251 | 83.5 | 53.91 | 94.33 |
| SW.3 | 95599 | 90165 | 22800394 | 252 | 87.43 | 54.38 | 94.32 |
| SW.4 | 53539 | 51569 | 12946262 | 251 | 77.67 | 54.05 | 96.32 |
| SW.5 | 88175 | 80172 | 20274521 | 252 | 90.00 | 53.93 | 90.92 |
| SW.6 | 82208 | 77446 | 19545796 | 252 | 86.34 | 52.23 | 94.21 |
| PD.1 | 53054 | 51328 | 13018547 | 253 | 80.30 | 54.25 | 96.75 |
| PD.2 | 96202 | 87746 | 22241650 | 253 | 77.37 | 54.2 | 91.21 |
| PD.3 | 84944 | 80178 | 20285137 | 253 | 87.40 | 54.64 | 94.39 |
| PD.4 | 84079 | 80150 | 20255107 | 252 | 88.20 | 52.19 | 95.33 |
| PD.5 | 83199 | 80112 | 20258414 | 252 | 87.21 | 54.41 | 96.29 |
| PD.6 | 85672 | 80224 | 20323729 | 253 | 81.59 | 53.76 | 93.64 |
| SO.1 | 82626 | 80300 | 20299482 | 252 | 88.59 | 54.26 | 97.18 |
| SO.2 | 83056 | 80085 | 20246596 | 252 | 88.68 | 54.26 | 96.42 |
| SO.3 | 59916 | 57609 | 14617416 | 253 | 78.54 | 54.52 | 96.15 |
| SO.4 | 75821 | 72673 | 18332235 | 252 | 86.37 | 49.81 | 95.85 |
| SO.5 | 81519 | 80084 | 20236102 | 252 | 82.59 | 48.69 | 98.24 |

Table A.2 Statistical table of Alpha diversity of intestinal flora in each group

| group | observed_species | shannon | simpson | chao1 | ACE | goods_coverage | PD_whole_tree |
| --- | --- | --- | --- | --- | --- | --- | --- |
| OS | 324 | 3.01 | 0.693 | 477.229 | 498.054 | 0.997 | 32.87 |
| SM | 477 | 3.736 | 0.809 | 649.791 | 662.602 | 0.996 | 38.196 |
| SW | 781 | 5.111 | 0.876 | 1147.889 | 1016.418 | 0.995 | 56.078 |
| PD | 836 | 5.685 | 0.894 | 1044.643 | 1065.389 | 0.995 | 61.015 |
| SO | 762 | 5.076 | 0.868 | 966.696 | 991.026 | 0.995 | 59.164 |

Table A.3 Figure 4A exact p-value

| group | chao1 (p_values) | observed_species (p_values) | PD_whole_tree (p_values) | shannon (p_values) |
| --- | --- | --- | --- | --- |
| OS_PD | 0.002670773 | 0.006288821 | 0.01224222 | 0.001806504 |
| OS_SM | 0.2363003 | 0.1719849 | 0.555852 | 0.2336862 |
| OS_SO | 0.01591518 | 0.01545918 | 0.04516391 | 0.03480959 |
| OS_SW | 0.002938646 | 0.006288821 | 0.03840127 | 0.01047016 |
| PD_SM | 0.003393159 | 0.000858664 | 0.002295748 | 0.000232826 |
| PD_SO | 0.5968324 | 0.5797546 | 0.8475569 | 0.4172063 |
| PD_SW | 0.5061457 | 0.6386591 | 0.5096251 | 0.2917685 |
| SM_SO | 0.05770547 | 0.06471117 | 0.06394962 | 0.1054372 |
| SM_SW | 0.00945456 | 0.02872562 | 0.03473224 | 0.02430496 |
| SO_SW | 0.3257296 | 0.9066737 | 0.7700151 | 0.9645992 |
